# Cardiomyocyte Nuclear Pleomorphism in a Mouse Model of Inherited Hypertrophic Cardiomyopathy

**DOI:** 10.3390/jcdd12110449

**Published:** 2025-11-19

**Authors:** Jamie R. Johnston, Isabella Leite Coscarella, Carson L. Rose, Yun Shi, Hosna Rastegarpouyani, Karissa M. Dieseldorff Jones, Jennifer M. Le Patourel, Feyikemi Ogunfuwa, Adriano S. Martins, Kathryn M. Crotty, Katherine M. Ward Molla, Tyler R. Reinoso, Taylor L. Waldmann, Jerome Irianto, Yue Julia Wang, Lili Wang, Björn C. Knollmann, Jose R. Pinto, Prescott Bryant Chase

**Affiliations:** 1 Department of Biomedical Sciences, College of Medicine, Florida State University, Tallahassee, FL 32306, USAjose.pinto@med.fsu.edu (J.R.P.); 2 Department of Biological Science, Florida State University, Tallahassee, FL 32306, USAchase@bio.fsu.edu (P.B.C.); 3 Institute for Molecular Biophysics, Florida State University, Tallahassee, FL 32306, USA; 4 Vanderbilt Center for Arrhythmia Research and Therapeutics, Vanderbilt University School of Medicine, Nashville, TN 37232, USA

**Keywords:** TNNC1, inherited cardiomyopathy, troponin, nucleus mechanics, hiPSC-derived cardiomyocytes

## Abstract

Mutations in genes encoding sarcomeric proteins are a common cause of cardiomyopathy and sudden cardiac death in humans. We evaluated the hypothesis that myofilament dysfunction is coupled to morphological and functional alterations of cardiomyocyte nuclei in a *Tnnc1*-targeted knock-in (*Tnnc1*-p.A8V) mouse model of hypertrophic cardiomyopathy (HCM). *Tnnc1* is the gene that codes for the isoform of the Ca^2+^-regulatory protein troponin C (cTnC) that is expressed in cardiomyocytes and slow skeletal muscle fibers and resides on thin filaments of sarcomeres in those muscles. This pathogenic mutation in a sarcomere gene alters many aspects of cardiomyocyte function, including sarcomere contractility, cytoplasmic Ca^2+^ buffering, and gene expression. Analysis of myocardial histological sections and isolated cardiomyocytes from adult *Tnnc1*-p.A8V mouse hearts revealed significantly smaller (cross-sectional area and volume) and rounder nuclei compared to those from age-matched, wild-type control mice. Changes in nuclear morphology could not be explained by differences in cardiomyocyte size or ploidy. Isolated wild-type and mutant cardiomyocyte nuclei, which are embedded centrally within myofibrils, undergo compression during contraction of the cardiomyocyte, indicating that during each heartbeat cardiomyocyte nuclei would be mechanically deformed as well as being exposed to elevated cytoplasmic Ca^2+^. Immunoblotting analysis indicated decreased nuclear localization of cardiac troponin C and decreased histone H4 expression in *Tnnc1*-p.A8V mouse hearts. Next, we investigated the influence of nucleocytoplasmic transport by immunofluorescence microscopy, and we could not confirm nuclear localization of cardiac troponin C in fixed myocardial tissue from adult mice. However, cardiac troponin C could be detected in healthy human-induced pluripotent stem cell-derived cardiomyocyte nuclei. We conclude that pathological myofilament dysfunction due to a pathogenic, cardiomyopathy-associated mutation can be linked to altered protein composition of cardiomyocyte nuclei and aberrant nuclear morphology.

## 1. Introduction

Pathogenic variants in genes encoding sarcomeric proteins represent a common cause of hypertrophic cardiomyopathy (HCM) and dilated cardiomyopathy (DCM) in humans, collectively referred to as sarcomeric cardiomyopathies [1]. HCM is clinically characterized by unexplained left ventricular hypertrophy with impaired relaxation, whereas DCM is characterized by left ventricular dilation with diminished systolic function [2]. Although the clinical trajectory of sarcomeric cardiomyopathies is variable, a significant proportion of patients often succumb to decompensated heart failure and sudden cardiac death [3]. The substantial morbidity and mortality associated with genetic cardiomyopathies underscores the need to better understand the molecular pathogenesis of these primary myocardial disorders, which could ultimately improve clinical management and treatment of genetically affected individuals [4,5].

The genetic landscape of cardiomyopathies is highly heterogeneous, encompassing mutations in more than 100 genes that encode proteins essential for cardiac structure and function [6]. These genes primarily involve elements of the sarcomere, cytoskeleton, desmosomes, ion channels, and nuclear envelope. Hypertrophic cardiomyopathy (HCM) most frequently arises from mutations in sarcomeric genes such as *MYH7* and *MYBPC3*, while dilated cardiomyopathy (DCM) is associated with a broader range of genes, including *TTN*, *LMNA*, and *DSP* [6]. Arrhythmogenic cardiomyopathy (ACM) typically results from mutations in desmosomal genes like *PKP2* and *DSG2*, whereas restrictive and left ventricular noncompaction cardiomyopathies often share overlapping genetic causes with HCM and DCM, leading to phenotypic and diagnostic overlap.

The genotype–phenotype relationship across cardiomyopathies is complex, influenced by variable penetrance, modifier genes, and environmental factors [6]. Advances in next-generation sequencing have expanded diagnostic precision, uncovering both rare pathogenic variants and variants of uncertain significance. Incorporating genetic testing into clinical evaluation now enables more accurate diagnosis, risk stratification, and familial screening. Nonetheless, significant challenges remain in variant interpretation and in elucidating the molecular mechanisms by which specific mutations lead to disease.

Integration of clinical and experimental findings has provided a strong basis for directly linking sarcomeric variants to cardiac dysfunction and heart failure [7]. Clinical examination by echocardiographic analysis of patient cohorts carrying a sarcomeric gene variant (‘genotype positive’) without overt structural remodeling (‘phenotype negative’) has suggested that sarcomere dysfunction represents an early biomechanical defect in genetically mediated cardiomyopathy [8]. Experiments leveraging biophysical and biochemical approaches have demonstrated that DCM variants are generally associated with diminished sarcomere contractility, while HCM variants are most often associated with enhanced sarcomere contractility and impaired relaxation [8,9]. A tension-based model grounded on experimental data was able to predict hypertrophic versus dilated heart growth for a given sarcomeric gene variant, further underscoring the significance of altered contractility in disease pathogenesis [10]. Mechanistically, these pathogenic variants have been shown to disrupt the highly coordinated regulatory activities of myofilament proteins and excitation-contraction coupling, thereby adversely impacting the magnitude and/or rate of tension generation [9,11]. Although the molecular consequences of pathogenic variants in sarcomeric proteins are well studied in the context of contractile regulation by Ca^2+^, the potential effects on cardiomyocyte nuclear morphology and mechanics are entirely unknown.

The cell nucleus is a mechanosensitive organelle that can respond dynamically to environmental stimuli [12,13]. Mechanical forces emanating from the extracellular space are transmitted through cytoskeletal networks and sensed by the nuclear lamina [14,15,16,17]. Several studies have focused on elucidating the molecular mechanisms of mechanosensing by the nucleus, which involve force-induced changes in chromatin organization and gene transcription [18,19,20,21,22]. This concept is of particular interest in the myocardium—a mechanically active tissue that is responsible for generating and thus experiences fluctuating pressures and tensile forces associated with each heartbeat [23]. Sarcomeres are physically linked to the nucleus via desmin-lamin intermediate filaments [24,25] and it has been demonstrated that cardiomyocyte nuclei undergo dynamic deformation during contraction [18,26,27,28,29]. It is therefore not surprising that variants in the genes encoding nuclear envelope proteins are associated with cardiovascular disease [30]. For example, pathogenic variants in the genes encoding A-type lamins [31,32,33], nesprins [34,35], and emerin [36] have been linked to DCM, cardiac conduction defects, and susceptibility to arrhythmias [37]. More recently, our group showed that pathogenic variants in *TNNT2*, both HCM and DCM, drive nuclear lamina remodeling in stem-cell-derived human cardiomyocytes [38]. Impaired nuclear mechanotransduction and nucleocytoskeletal instability have been implicated in the disease pathogenesis [17,23]. Mechanistically, disruption of force transmission by these pathogenic variants can lead to impairments in signaling, nucleocytoplasmic transport, and transcriptional/epigenetic regulation of gene expression. Indeed, gene expression and protein synthesis in the cell might be partially determined by the physical properties of the nucleus itself [39,40], and disrupted nucleo-cytoplasmic transport is associated with morphological alterations of nuclei in various cell types [41]. Therefore, intact mechanosensing and maintenance of the cardiomyocyte nuclear architecture appear to be critical for cardiac homeostasis.

Herein, we investigated cardiomyocyte nuclei in a previously established *Tnnc1* knock-in mouse model that encodes a pathogenic alanine to valine substitution at amino acid residue 8 (A8V) in the sarcomeric protein, TNNC1 [42]. These mice reproducibly exhibit the clinical and molecular characteristics of HCM, such as hyperdynamic systolic function, diastolic dysfunction, increased myofilament Ca^2+^ sensitivity, and altered gene expression, thereby providing a robust model to study the pathogenic mechanisms of sarcomeric cardiomyopathy [42,43,44,45]. As noted in the initial report on this mouse model of HCM, there is some phenotypic overlap with restrictive cardiomyopathy, which was also observed in the proband of the initial study [43] and in subsequent patient studies [46,47]. Examination of cardiomyocyte nuclei in myocardial tissue as well as isolated cells from Tnnc1-p.A8V mice reveals alterations in nuclear structure and function. We propose that cardiomyocyte nuclear remodeling might be linked to the pathogenesis of HCM.

## 2. Materials and Methods

### 2.1. Animal Procedures

*Tnnc1* homozygous (*Tnnc1*^A8V/A8V^), heterozygous (*Tnnc1*^A8V/WT^), control (*Tnnc1*^WT/WT^) knock-in mice were generated as previously described [42]. When possible, but not in all circumstances, littermates were used as controls. For terminal procedures, animals were euthanized with isoflurane followed by rapid cervical dislocation. The animal protocol was approved by the Florida State University Animal Care and Use Committee (ACUC Protocol #1736) and performed in accordance with the Guide for the Care and Use of Laboratory Animals outlined by the National Institutes of Health (NIH). Mice were housed in a temperature-controlled vivarium on a 12:12 h light/dark cycle with ad libitum access to water and normal chow.

### 2.2. Histology

Hearts from *Tnnc1*^A8V/WT^ and *Tnnc1*^WT/WT^ 18-month-old mice of either sex were excised and immersed in 10% formaldehyde; longitudinal tissue sectioning and hematoxylin and eosin (H&E) staining was carried out by IDEXX Inc. (Boston, MA, USA), essentially as described previously [42] and as illustrated in Dieseldorff Jones et al. [45]. Briefly, tissues were trimmed into the Excelsior AS Tissue Processor (Epredia, Kalamazoo, MI, USA). The tissues were processed with graded alcohols dehydration, xylene for clearing, paraffin for infiltration. Processed tissues were transferred to the Sakura Tissue-Tek for embedding. The embedded tissues were sectioned into paraffin blocks at 3–5 µm thickness using the HM 355S Microtome (Epredia, Kalamazoo, MI, USA) and then mounted onto charged glass slides. Tissue slides were stained with hematoxylin and eosin (H&E) using the Tissue-Tek Prisma Plus Automated Slide stainer (Sakura Finetek USA, Torrance, CA, USA) with a Tissue-Tek 4740 film coverslipper (Sakura Finetek USA, Torrance, CA, USA). Heart sections were imaged on an Olympus BX61 microscope using a 40×/0.65 Plan Apo S objective (Olympus Corporation of the Americas, Center Valley, PA, USA) and digital images were recorded with an Olympus DP71 camera (Olympus Corporation of the Americas, Center Valley, PA, USA). All geometric measurements of cardiomyocyte nuclei samples were performed in a blinded manner with respect to mouse genotype.

### 2.3. Cardiomyocyte Isolation

Cardiomyocytes were harvested from 2 to 4-month-old mice of either sex using the Langendorff method, essentially as described [42]. In brief, *Tnnc1*^WT/WT^ and *Tnnc1*^A8V/A8V^ mice were injected intraperitoneally with 0.5 mL heparin diluted to 100 IU/mL in PBS and, 15 min post-injection, the mice were euthanized by cervical dislocation. Hearts were excised rapidly and placed in a cold chamber containing 120 mmol/L, NaCl, 5.4 mmol/L KCl, 1.2 mmol/L MgSO_4_, 1.2 mmol/L NaH_2_PO_4_, 5.6 mmol/L glucose, 20 mmol/L NaHCO_3_, 20 mmol/L 2,3-butanedione monoxime (BDM), and 5 mmol/L taurine (Sigma, St. Louis, MO, USA), pH 7.4 and gassed with 95% O_2_/5% CO_2_. After exposure of the aortic arch, the hearts were connected to a 22G cannula, which was inserted into the descending aortic arch, and perfused with Ca^2+^-free buffer to remove remaining blood in the coronary arteries and veins. To isolate individual cardiomyocytes, the hearts were perfused with digestion buffer containing 1 mg/mL collagenase type II (Worthington Biochemical, Lakewood, NJ, USA) and 0.1 mg/mL protease type XIV (Sigma, St. Louis, MO, USA) for 5 min. The cardiomyocytes were harvested from the digested cardiac tissue by gentle dissociation, achieved by passage through a plastic pipette. Cardiomyocytes were subsequently transferred to a conical tube and filtered through a cell strainer. Ca^2+^ was gradually reintroduced to the culture medium in four steps (15 min each): 0.25 mmol/L, 0.50 mmol/L, 0.75 mmol/L and 1 mmol/L.

### 2.4. Confocal Microscopy of Adult Mouse Cardiomyocytes

Living cardiomyocytes were simultaneously stained for 45 min with live cell staining NucBlue^®^ Live reagent (Molecular Probes-Thermo Fisher Scientific, Waltham, MA, USA) following the manufacturer instructions to label DNA, and 10 μmol/L Fluo-5N/AM (Molecular Probes-Thermo Fisher Scientific, Waltham, MA, USA) which is a membrane permeant, low affinity Ca^2+^ dye; staining was followed by 45 min wash at 37 °C. Cardiomyoyctes were then transferred to 35 mm glass bottom Petri dishes coated with laminin, and maintained in 1.8 mmol/L Ca^2+^ medium for confocal imaging. Laser-scanning fluorescence confocal microscopy was performed on an Andor Revolution Spinning Disk laser confocal microscope (Andor USA, Concord, MA, USA) for live cell imaging. The Andor confocal was equipped with an Imaging Yokogawa (Yokogawa Corporation of America, Sugar Land, TX, USA) automated 5000 rpm spinning disk and a Nikon Eclipse Ti Microscope (Nikon Instruments Inc., Melville, NY, USA) with perfect focus system. Imaging was accomplished with either a 60× or 100× oil immersion objective. Fluo-5N was excited using the 488 nm line of the argon ion laser and emission was recorded at 530 nm. NucBlue^®^ Live reagent dye was excited using 405 nm and emission was recorded at 435 nm. Confocal images of cardiomyoyctes were acquired as image pairs, time series, or serial Z-stacks of interlaced images for the NucBlue^®^ Live reagent channel (nuclei) and the Fluo-5N channel (Ca^2+^). Z-stacks were recorded with z-spacing of 0.40–0.51 µm. Digital images were saved as series of 16-bit tagged image file format (TIFF) files for further processing. Imaging of isolated cardiomyocytes was also carried out in fluorescence mode on an Olympus BX61 microscope (Olympus Corporation of the Americas, Center Valley, PA, USA).

### 2.5. Image Analysis of Adult Mouse Cardiomyocytes

All geometric measurements of cardiomyocyte nuclei samples were performed in a blinded manner with respect to mouse genotype. Images were analyzed with ImageJ Version 1.48 [48]. The 16-bit TIFF z-stack files obtained via fluorescence confocal microscopy were utilized for 2D analysis of images and 3D reconstruction and analysis of isolated, mouse cardiomyocytes and their nuclei. Images of a stage reticle were used to calibrate the x- and y-dimensions, and the manufacturer’s calibration stored with the data was used for the z dimension. Z-stacks were split into two sub-stacks corresponding to the two channels—NucBlue^®^ Live nuclear stain and Fluo-5N—using the Substack Maker Plus plugin [49]. The regions of interest (ROIs) in each image were generated using ImageJ’s Image > Adjust > Threshold function to highlight the cardiomyocyte (Fluo-5N) or nucleus/nuclei (NucBlue^®^ Live) from their respective stacks; non-highlighted regions within the ROIs were filled using ImageJ’s Process > Binary > Fill Holes function. Each ROI was selected using the wand (tracing) tool in ImageJ’s toolbar, and length, width and cross-sectional area were obtained using the Analyze > Measure function. Nucleus volume was obtained from z stacks of known z spacing by 3-D integration over all image slices of an individual nucleus. ZEN 3.1 (Blue/Black) software (Zeiss Microscopy Solutions, White Plains, NY, USA) was used for analysis of confocal images of a subset of isolated, mouse cardiomyocyte images and hiPSC-CM images.

**Figure 1:**
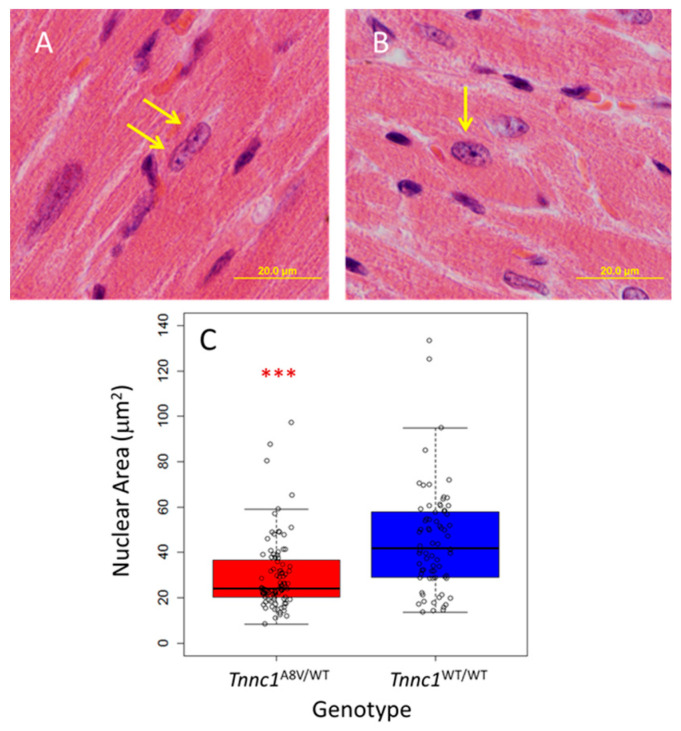
Cardiomyocyte nuclei areas of *Tnnc1*^A8V/WT^ are smaller than *Tnnc1*^WT/WT^. Representative micrographs of H&E stained, fixed tissue slices from two 18-month-old *Tnnc1*^A8V/WT^ (**A**) and two *Tnnc1*^WT/WT^ (**B**) mouse hearts. Fixed hearts were longitudinally sectioned as illustrated in Dieseldorff Jones et al. [45]. Example nuclei that were included in the analysis are indicated by arrows. Boxplot (**C**) summary of nuclei areas (*** *p* < 0.001 compared to *Tnnc1*^WT/WT^). *Tnnc1*^WT/WT^ (*n* = 72 nuclei), *Tnnc1*^A8V/WT^ (*n* = 92 nuclei). *p* < 0.001, two-sided, Welch Two Sample *t*-test. Data are presented as the median with 1st and 3rd quartile ranges.

**Figure 2:**
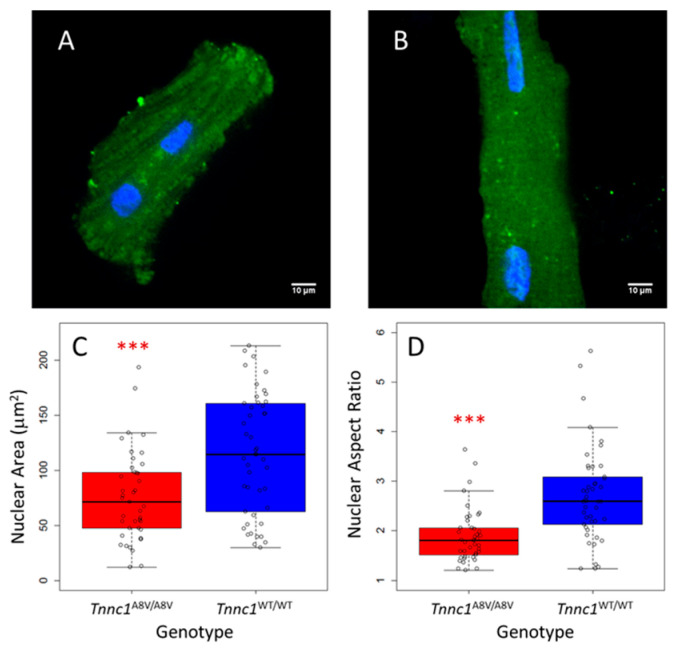
Cardiomyocyte nuclei of *Tnnc1*^A8V/A8V^ are smaller and rounder than *Tnnc1*^WT/WT^. Representative confocal micrographs of relaxed, living cardiomyocytes, isolated from five 2–4 month-old *Tnnc1*^A8V/A8V^ (**A**) or three *Tnnc1*^WT/WT^ (**B**) animals and stained with NucBlue (blue) and Fluo-5N AM (green). (**C**) Boxplot summary of nuclei areas and (**D**) nuclei length/width ratios (*** *p* < 0.001 compared to *Tnnc1*^WT/WT^, two-sided, Welch Two Sample *t*-test). *Tnnc1*^WT/WT^ (*n* = 46 nuclei), *Tnnc1*^A8V/A8V^ (*n* = 41 nuclei). Data are presented as the median with 1st and 3rd quartile ranges.

**Figure 3:**
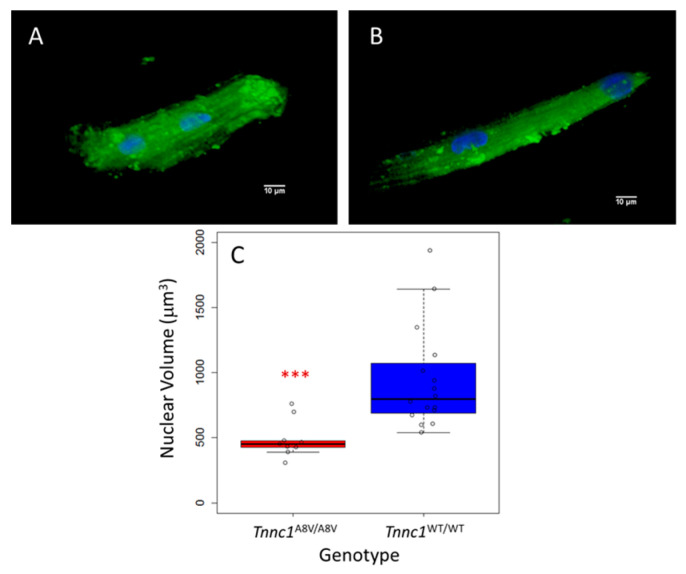
Cardiomyocyte nuclei of *Tnnc1*^A8V/A8V^ homozygotes have smaller volumes than *Tnnc1*^WT/WT^. Representative composites from confocal z-stacks of relaxed, living cardiomyocytes, isolated 2–4-month-old *Tnnc1*^A8V/A8V^ (**A**) or *Tnnc1*^WT/WT^ (**B**) animals and stained with NucBlue (blue) and Fluo-5N AM (green) (See Section 2.4). Boxplot (**C**) summary of nuclei volumes (*** *p* < 0.001 compared to *Tnnc1*^WT/WT^, two-sided, Welch Two Sample *t*-test). *Tnnc1*^WT/WT^ (*n* = 16 nuclei), *Tnnc1*^A8V/A8V^ (*n* = 9 nuclei). Data are presented as the median with 1st and 3rd quartile ranges.

**Figure 4:**
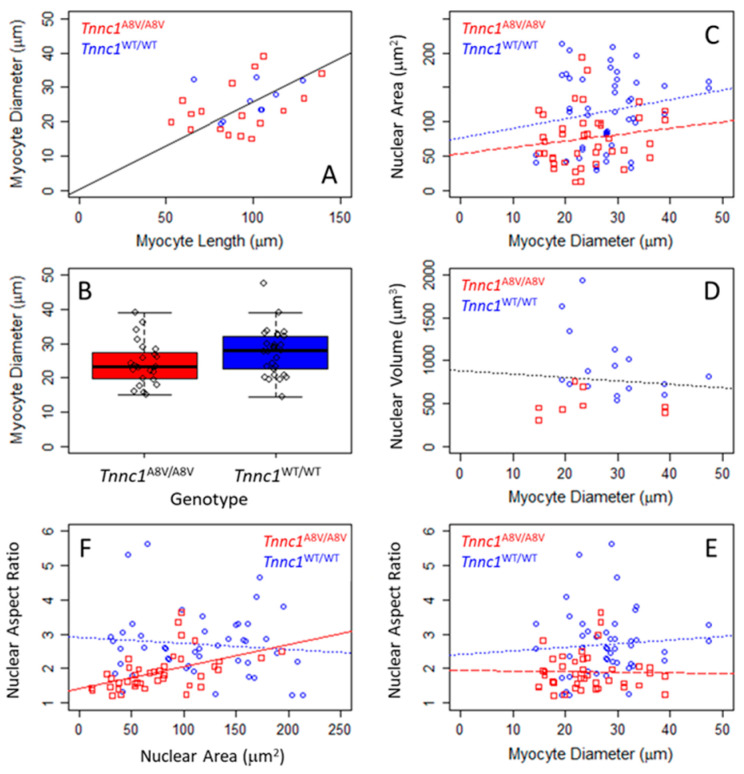
Nucleus dimensions are not related to cardiomyocyte dimensions. (**A**) In a subset of relaxed, living, isolated cardiomyocytes where both cell length and diameter could be measured from the Fluo-5N signal, diameter was 25–26% of cell length for both *Tnnc1*^A8V/A8V^ (red squares) and *Tnnc1*^WT/WT^ (blue circles) 2–4-month-old mice. The regression line (black) corresponds to the fit to all data points and was constrained to pass through the origin (multiple R^2^ = 0.928). (**B**) Boxplot summary of cardiomyocyte diameters. *Tnnc1*^A8V/A8V^ diameters (red) were not statistically different (*p* > 0.05, two-sided, Welch Two Sample *t*-test) from *Tnnc1*^WT/WT^ (blue). Nucleus area (**C**), volume (**D**), and aspect ratio (**E**) are not related to myocyte diameter for *Tnnc1*^A8V/A8V^ (red squares and lines) or *Tnnc1*^WT/WT^ (blue circles and lines) cardiomyocytes. In all three panels (**C**–**E**), the regression slopes were not significantly different from 0 (dotted lines, *p* > 0.05, two-sided, Welch Two Sample *t*-test); note that the regression line in panel (**D**) corresponds to the fit to all data points because of the small number of nucleus volume measurements. (**F**) Cardiomyocyte nucleus shape (aspect ratio) is related to nucleus area for *Tnnc1*^A8V/A8V^ (red squares and solid line) but not *Tnnc1*^WT/WT^ (blue circles and dotted line). *Tnnc1*^WT/WT^ (*n* = 24 myocytes), *Tnnc1*^A8V/A8V^ (*n* = 29 myocytes). Data in panel (**B**) are presented as the median with 1st and 3rd quartile ranges.

Except for assessing the correlation between paired nuclei in binucleated cardiomyocytes (Supplementary Material, Figure S2), we included all nuclei, independent of nuclearity, analyzed from all cardiomyocytes for measurements of nuclear size (area and volume) (Figure 1, Figure 2, Figure 3 and Figure 4). Isolated, living cardiomyocytes sometimes contracted during confocal imaging (Supplementary Material, Figures S3 and S4). For the measurements shown in Figure 2, Figure 3 and Figure 4 and (Supplementary Material, Figure S2), cardiomyocytes were only included if they were at rest (i.e., not contracting). In addition to the primary analysis on resting cardiomyocytes, we were able to obtain confocal time series for some contracting myocytes (Supplementary Material, Figures S3 and S4); we analyzed nuclear and cardiomyocyte dimensions from cardiomyocytes where (i) the ends of the cell were clearly visible so that myocyte length could be determined; (ii) the time series begins while the myocyte was at the resting (diastolic) length; and (iii) at least one nucleus, and preferably both if the cardiomyocyte was binucleated, remained in the same focal plane throughout the time series (i.e., only horizontal, and little or no vertical motion during contraction).

### 2.6. Preparation of Isolated Nuclei and Flow Cytometry

Nuclei were isolated from mouse ventricular tissue of *Tnnc1*^A8V/A8V^ (*n* = 3) and *Tnnc1*^WT/WT^ (*n* = 3) mice, mixed sexes, 4–6 months of age, following the protocol of Bergmann and Jovinge [50] essentially as described but with the addition of 1% bovine serum albumin (BSA) to the nuclear storage buffer (NSB: 0.43 M sucrose, 70 mM KCl, 2 mM MgCl_2_, 10 mM Tris–HCl (pH 7.2), and 5 mM EGTA). Briefly, isolated nuclei were labeled overnight at 4 °C with rabbit anti-PCM-1 (Sigma, St. Louis, MO, USA, HPA023374) or isotype rabbit IgG (Abcam, Waltham, MA, USA, ab37415), both used at 8 µg/mL final concentration in NSB (0.44 mol/L sucrose, 10 mmol/L Tris-HCl pH 7.2, 70 mmol/L KCl, 1.5 mmol/L spermine, 10 mmol/L MgCl_2_, 1% BSA). The following day, nuclei were washed 2× with NSB and incubated for 1 h at 4 °C with anti-rabbit secondary antibody conjugated to Alexa Fluor-488 (Thermo Fisher, Waltham, MA, USA, at 1:1000 in NSB. Nuclei were washed 2×, resuspended in NSB, and incubated with NucBlue™ Hoechst 33342 (Life Technologies, Carlsbad, CA, USA, R37605) at room temperature for 20 min. Flow cytometry was performed on a BD FACSAria Special Order System (Becton Dickinson, Franklin Lakes, NJ, USA) and BD FACSDiva 8.0.1 software (Becton Dickinson) was used for data analysis and plotting. Alexa Fluor 488–conjugated secondary antibodies were excited with the 488 nm laser, and fluorescence emission was collected using a 530/30 bandpass filter (FITC channel). NucBlue™ Hoechst 33342–stained nuclei were excited with the 355 nm UV laser, and fluorescence emission was collected using a 450/50 bandpass filter (DAPI channel). Doublet discrimination was performed. At least 17,000 PCM-1^+^ events were recorded for each group.

### 2.7. Generation of Human iPSC-CMs

Human induced pluripotent stem cells (hiPSCs) were generated from healthy volunteers as previously described [51]. Cardiac induction of hiPSCs to hiPSC-derived cardiomyocytes hiPSC-CMs was carried out by small molecule-based cardiac differentiation, CHIR99021 (Selleck Chemicals, Houstin, TX, USA) and IWR-1 (Sigma), as previously described [51,52]. Briefly, (hiPSC-CMs) were produced using a small molecule-based cardiac differentiation protocol. Briefly, hiPSCs at passages greater than 20 were split at a 1:12 ratio using 0.5 mmol/L EDTA (Life Technologies) in D-PBS without Ca^2+^ or Mg^2+^ (Life Technologies), as described above. Cells were cultured for four days until reaching approximately 80% confluence, designated as day 0. At this point, the medium was switched to basal RPMI 1640 (11875, Life Technologies) supplemented with B27 without insulin (A1895601, Life Technologies) and 6 µmol/L CHIR99021 (Selleck Chemicals). On day 2, the medium was replaced with RPMI 1640 plus B27 without insulin, omitting CHIR99021. On day 3, basal RPMI 1640 with B27 without insulin was supplemented with 5 µmol/L IWR-1 (Sigma). The medium was refreshed on day 5 with RPMI 1640 plus B27 without insulin and subsequently every other day until day 10. On day 10, cells were transitioned to a metabolic selection medium consisting of glucose-free RPMI 1640 (11879, Life Technologies) with B27 without insulin. On day 12, the medium was switched to RPMI 1640 (11875, Life Technologies) containing 2% B27 supplement (Invitrogen, Carlsbad, CA, USA) and 1% Penicillin-Streptomycin (Life Technologies) until cell dissociation. Spontaneous contractions were first observed around day 7. HiPSC-CMs were stored in liquid nitrogen until use for experiments.

### 2.8. Confocal Imaging of hiPSC-CMs

hiPSC-CMs were cultured in RPMI 1640 media (GenClone), supplemented with 2% B-27 (Thermo Fisher, Waltham, MA, USA) and 1% penicillin and streptomycin (Corning, Corning, NY, USA). Prior to cell seeding, the tissue culture surface was treated with 1:200 Matrigel (Corning) solution for 30 min. For immunostaining, hiPSC-CMs were fixed in 4% formaldehyde (Electron Microscopy Sciences, Hatfield, PA, USA) for 15 min, permeabilized by 0.5% Triton X-100 (Sigma) for 10 min, blocked by 5% BSA (VWR), and incubated overnight in primary antibodies against cardiac troponin C (Genetex, Irvine, CA, USA, GTX 33061) and lamin-A/C (Cell Signaling, Danvers, MA). The primary antibodies were then tagged with the corresponding secondary antibodies (Thermo Fisher) for 1.5 h. For F-actin staining, 100 ng/mL TRITC-phalloidin (Sigma, St. Louis, MO, USA) was also added to the secondary antibody solution to label cytosolic (sarcomeric) F-actin. DNA was stained with 8.0 µmol/L Hoechst 33342 (Thermo Fisher, Waltham, MA, USA) for 15 min. Confocal imaging was using a Leica TCS SP8 system with a 63×/1.4 NA oil-immersion objective. Image processing was performed using ImageJ [48].

### 2.9. Immunoblotting

Protein concentrations of cytoplasmic and nuclear extracts, prepared as previously described [53], were determined using Pierce 660 nm protein assay reagent following the manufacturer’s instructions (ThermoFisher) and diluted with Laemmli sample buffer (Bio-Rad, Hercules, CA). Myofibrils were isolated as previously described [54]. Equal amounts of protein were resolved on a 15% SDS-PAGE gel and then transferred at 90 V for 90 min at 4 °C to a 0.45 µm nitrocellulose membrane (Amersham Biosciences, Woburn, MA, USA) for immunoblotting. The membrane was blocked for 1 h at room temperature in blocking buffer (5% nonfat dry milk in PBS-T plus 0.2% Tween-20). After 3 (5 min) washes, membranes were incubated overnight in blocking buffer with primary antibodies against cardiac troponin C (Santa Cruz, Dallas, TX, sc-52265), GAPDH (Santa Cruz, Dallas, TX, sc-25778), lamin A/C (Santa Cruz, Dallas, TX, sc-376248), cardiac troponin I (Developmental Studies Hybridoma Bank, Iowa City, IA, USA, TI-1), and histone H4 (courtesy of Prof. Akash Gunjan, Florida State University College of Medicine). The following day, membranes were washed as described above and incubated for 1 h at room temperature in blocking buffer containing secondary antibodies anti-mouse IRDye 680RD and IRDye 800CW at 1:20,000 dilution (LI-COR Biosciences, Lincoln, NE, USA). Membranes were washed again and then imaged using the Odyssey infrared imaging system (LI-COR Biosciences, Lincoln, NE, USA). ImageJ was used for densitometric quantification of the protein bands [48]. Loading controls are indicated in the respective figure legends.

## 3. Statistical Analysis

Statistical analyses, including regressions, were performed using R (V3.3.0–3.4.0) and GraphPad Prism (V8.3.0). Note that box-and-whisker plots illustrate the median (line), the 1st and 3rd quartiles (bottom and top of box, respectively), +1.58 IQR/sqrt(*n*) (whiskers; this is approx. 95% confidence interval), and any data outside of this range are plotted as individual points. The specific statistical test used to compare a data set is stated in each figure legend. Data are reported as mean ± SD, otherwise as indicated in the figure legends or text. Sample size is denoted as “*n*” and is reported in each figure legend. *p*-values below 0.05 were considered statistically significant.

## 4. Results

### 4.1. Tnnc1-p.A8V Mice Exhibit Smaller and Rounder Cardiomyocyte Nuclei

To explore the hypothesis that cardiomyocyte nuclear size is altered in sarcomeric cardiomyopathy, we used our previously established Tnnc1-p.A8V knock-in mouse model [42]. Homozygous mice (*Tnnc1*^A8V/A8V^) display the characteristic features of HCM by 3 months of age, and the heterozygous mice (*Tnnc1*^A8V/WT^) exhibit cardiac restriction by 14 months of age. We first examined cardiomyocyte nuclei in fixed myocardial tissue sections stained with hematoxylin and eosin from age-matched *Tnnc1*^WT/A8V^ and wild-type control (*Tnnc1*^WT/WT^) mice at 18 months of age. We were careful to select only nuclei that were within clearly defined cardiomyocyte borders and measured the area of each nucleus. Representative images of the cardiomyocyte nuclei, indicated by yellow arrows, are shown in Figure 1A (*Tnnc1*^A8V/WT^) and Figure 1B (*Tnnc1*^WT/WT^). Cardiomyocyte nucleus areas for *Tnnc1*^A8V/WT^ cardiomyocytes (29.6 ± 15.9 μm^2^) were, on average, 65.7% of *Tnnc1*^WT/WT^ (45.1 ± 23.0 μm^2^) (Figure 1C); this difference was statistically significant (*p* < 0.001). These results suggest that, at the tissue level, cardiomyocyte nuclei in a mouse model of HCM are smaller compared to healthy mice.

Next, we examined nuclei in freshly isolated cardiomyocytes to avoid potential artifacts associated with fixation/sectioning of tissue and to ensure unequivocal identification of myocyte nuclei. Cardiomyocytes were isolated from age-matched homozygous mutant (*Tnnc1*^A8V/A8V^) and *Tnnc1*^WT/WT^ mice and imaged by confocal microscopy. Younger (2–4-month-old) mice were chosen for this analysis and all subsequent analyses to exclude the potential for confounding effects of aging. Representative cardiomyocytes are shown in Figure 2A (*Tnnc1*^A8V/A8V^) and Figure 2B (*Tnnc1*^WT/WT^). Nucleus areas for *Tnnc1*^A8V/A8V^ cardiomyocytes (76.2 ± 40.9 μm^2^) were, on average, 66.3% of *Tnnc1*^WT/WT^ nuclei (114.8 ± 54.7 μm^2^) (Figure 2C); this difference was statistically significant (*p* < 0.001). These results suggest that cardiomyocyte nuclei in a mouse model of HCM are smaller compared to healthy mice at the cellular level. The nuclei of *Tnnc1*^WT/WT^ cardiomyocytes shown in Figure 2B not only have a significantly larger area, but they also appear to be more elongated than the *Tnnc1*^A8V/A8V^ cardiomyocyte nuclei shown in Figure 2A. We therefore examined whether this was generally true for the population of nuclei examined. Analysis of nucleus shape (aspect ratio, or length/width ratio) indicated that *Tnnc1*^A8V/A8V^ cardiomyocyte nuclei (1.9 ± 0.6) were significantly rounder (i.e., less elongated) than *Tnnc1*^WT/WT^ nuclei (2.7 ± 1.0) (*p* < 0.001) (Figure 2D).

In a subset of isolated, living cardiomyocytes, we measured nucleus volumes from confocal z stacks to ensure that cross-sectional area (Figure 1 and Figure 2) accurately reflects the dimensions of the entire nucleus. Representative cardiomyocyte images reconstructed from z-stacks are shown in Figure 3A (*Tnnc1*^A8V/A8V^) and Figure 3B (*Tnnc1*^WT/WT^). Nucleus volumes for *Tnnc1*^A8V/A8V^ cardiomyocytes (490.1 ± 145.5 μm^3^) were, on average, significantly smaller compared to *Tnnc1*^WT/WT^ cardiomyocytes (942.0 ± 394.9 μm^3^) (*p* < 0.001) (Figure 3C). We next sought to determine whether the differences in nucleus volumes between cardiomyocytes from *Tnnc1*^A8V/A8V^ V mice and *Tnnc1*^WT/WT^ mice (Figure 3) are consistent with the difference in nucleus cross-sectional areas (Figure 1 and Figure 2). Combining the simplifying assumption of spherical geometry with the observation that the average volume of *Tnnc1*^A8V/A8V^ cardiomyocyte nuclei was 52.0% of *Tnnc1*^WT/WT^ nuclei (Figure 3), we would expect the cross-sectional area of *Tnnc1*^A8V/A8V^ cardiomyocyte nuclei to be (0.52)^2/3^, or 64.7%, of *Tnnc1*^WT/WT^ nuclei; this compares favorably with the observed values of 65.7% (Figure 1) and 66.3% (Figure 2).

In rodents, cardiomyocytes are predominantly binucleated [55,56,57]. We therefore explored the possibility of a correlation between the sizes of paired nuclei within single cardiomyocytes. In agreement with prior reports, we found that most cardiomyocytes from *Tnnc1*^WT/WT^ and *Tnnc1*^A8V/A8V^ mice were binucleated (Supplementary Material, Figure S1. For binucleated cardiomyocytes where both nuclei were well-defined, we designated nucleus #1 as the nucleus with the larger cross-sectional area, and thus nucleus #2 was the smaller of the two nuclei (Supplementary Material, Figure S2). Paired, one-tailed (nucleus #1 > nucleus #2) *t*-tests indicated that both nucleus area (*p* < 0.001) and volume (*p* < 0.01) were significantly different between the paired nuclei; note that for this analysis, the volume data for *Tnnc1*^A8V/A8V^ and *Tnnc1*^WT/WT^ were combined due to small sample sizes. To assess the magnitude of the difference between paired nuclei, we used linear least squares regression analysis where the regression was constrained to pass through the origin. Areas (Supplementary Material, Figure S2A) and volumes (Supplementary Material, Figure S2B) for nucleus pairs in binucleated cardiomyocytes were strongly correlated (multiple R^2^ > 0.934). For *Tnnc1*^WT/WT^ cardiomyocytes, the area of the smaller nucleus (nucleus #2) was 0.86 ± 0.02 (slope parameter estimate ± SE) relative to the larger nucleus (Supplementary Material, Figure S2A). For *Tnnc1*^A8V/A8V^ cardiomyocytes, the area of the smaller nucleus (nucleus #2) was 0.69 ± 0.04 (slope parameter estimate ± SE) relative to the larger nucleus #1 (Supplementary Material, Figure S2A). The volume of the smaller nucleus (nucleus #2) was 0.66 ± 0.05 (slope parameter estimate ± SE) relative to the larger nucleus (Supplementary Material, Figure S2B).

We also examined the relationship between the shapes of the two nuclei. Paired, two-tailed *t*-tests indicated that nucleus aspect ratio (length/width) was not significantly different between nucleus pairs for Tnnc1WT/WT cardiomyocytes (*p* > 0.05), but was significantly different for *Tnnc1*^A8V/A8V^ (*p* < 0.01) (Supplementary Material, Figure S2C); we note that the designation of nucleus #1 and #2 according to nucleus area, in combination with the correlation of nucleus shape and size for *Tnnc1*^A8V/A8V^ only (but not *Tnnc1*^WT/WT^) cardiomyocytes (Figure 4), may contribute to the statistical significance (or lack thereof) of these comparisons. To assess the magnitude of any difference between paired nuclei, we used nonlinear regression analysis where the regression was a straight line constrained to pass through the point (1, 1). For *Tnnc1*^WT/WT^ cardiomyocytes, the aspect ratio of the smaller nucleus (nucleus #2) was 0.77 ± 0.11 (slope parameter estimate ± SE) relative to the larger nucleus (Supplementary Material, Figure S2C). For *Tnnc1*^A8V/A8V^ cardiomyocytes, the aspect ratio of the smaller nucleus (nucleus #2) was 0.66 ± 0.07 (slope parameter estimate ± SE) relative to the larger nucleus (Supplementary Material, Figure S2C). The slopes of both regressions were significantly different from 0 (*p* < 0.001). The smaller value of the slope for *Tnnc1*^A8V/A8V^ is in concert with the data in Figure 2D that indicate *Tnnc1*^A8V/A8V^ nuclei exhibit rounder morphology (i.e., aspect ratio ~ 1). Taken together, these results suggest that in binucleated cardiomyocytes, the sizes of the paired nuclei are correlated, but one nucleus is significantly larger than the other in myocytes from both *Tnnc1*^WT/WT^ and *Tnnc1*^A8V/A8V^ mice.

### 4.2. Smaller Tnnc1-p.A8V Cardiomyocyte Nuclei Cannot Be Explained by Differences in Cell Size or DNA Content

Under physiological conditions, nucleus size in interphase tends to scale with cell size in a variety of cells and organisms, thus maintaining a constant nuclear-to-cytoplasmic volume ratio (N/C) [58]. Based on our observations that *Tnnc1*^A8V/A8V^ cardiomyocyte nuclei are smaller than *Tnnc1*^WT/WT^ nuclei, we next asked whether cell size could account for this difference. Figure 4A informs us that cell diameter—which could be measured from images of every cardiomyocyte—suffices as a proxy for cardiomyocyte size. The linear least squares regression, constrained to pass through the origin, on all data in Figure 4A indicates that cardiomyocyte diameter is ~26% of cell length (multiple R^2^ = 0.928). Considering all living, isolated cardiomyocytes examined in this study, the diameter of *Tnnc1*^A8V/A8V^ cardiomyocytes (24.2 ± 6.4 μm) was not statistically different (*p* > 0.05) from *Tnnc1*^WT/WT^ (27.5 ± 6.8 μm); this result alone suggests that the differences in nucleus size (Figure 1, Figure 2 and Figure 3) are not likely due to differences in cardiomyocyte size. Furthermore, we did not find statistically significant relationships between area (Figure 4C), volume (Figure 4D) or shape (Figure 4E) of individual nuclei and the diameter of the cardiomyocyte in which it was located; slopes for all regressions in Figure 4C–E were not different from 0 (*p* > 0.05), with multiple R^2^ < 0.176. Therefore, we conclude that differences in nucleus size cannot be ascribed to differences in cardiomyocyte size.

The results in Figure 2 suggest that larger cardiomyocyte nuclei may be more elongated, so we next asked whether the aspect ratio of a cardiomyocyte nucleus is related to its size. No significant relationship exists for *Tnnc1*^WT/WT^ nuclei (Figure 4F, circles and dotted line); the *Tnnc1*^WT/WT^ regression slope in Figure 4F was not statistically different from 0 (*p* > 0.05), with regression multiple R^2^ = 0.010. In contrast, *Tnnc1*^A8V/A8V^ cardiomyocyte nucleus aspect ratio was related to nucleus area (Figure 4F, squares and solid line); the *Tnnc1*^A8V/A8V^ regression slope in Figure 4F was significantly different from 0 (*p* < 0.01), with regression multiple R^2^ = 0.233. It is therefore possible that *Tnnc1*^A8V/A8V^ nuclei may be rounder (Figure 2D) because they are smaller (Figure 2A–C and Figure 3), although the absence of an equivalent relationship for *Tnnc1*^WT/WT^ nuclei renders uncertain the biological significance of this observation.

**Figure 5:**
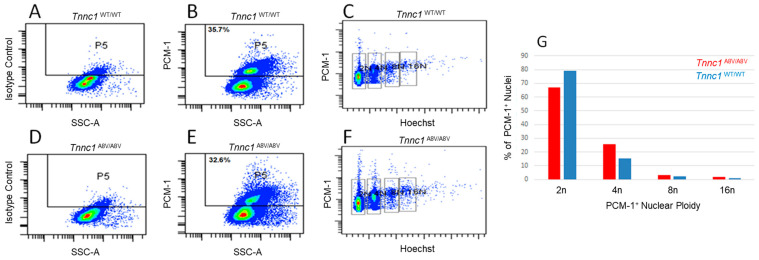
Representative flow cytometry plots of purified cardiomyocyte (PCM-1^+^) nuclei isolated from 4 to 6-month-old *Tnnc1*^WT/WT^ mice (**A**–**C**) and *Tnnc1*^A8V/A8V^ mice (**D**–**F**). Nuclei were immunolabeled with IgG isotype control (**A**,**D**) or anti-PCM-1 (**B**,**E**) followed by a secondary antibody conjugated to Alexa-488. (**C**,**F**) PCM-1^+^ versus Hoechst 33342 for assessment of cardiomyocyte nuclear ploidy. Doublet discrimination was performed. (**G**) Percent (%) of diploid (2n), tetraploid (4n), octoploid (8n), and hexadecaploid (16n) cardiomyocyte nuclei populations isolated from *Tnnc1*^WT/WT^ and *Tnnc1*^A8V/A8V^ V mouse cardiac ventricular tissue *Tnnc1*^WT/WT^ (*n* = 3 pooled hearts), *Tnnc1*^A8V/A8V^ (*n* = 3 pooled hearts).

Based on the observations that *Tnnc1*^A8V/A8V^ cardiomyocyte nuclei were approximately half the volume of *Tnnc1*^WT/WT^ nuclei and that this difference could not be explained by cell size, we next asked whether *Tnnc1*^A8V/A8V^ mice may have lower DNA content. To address this question, we carried out ploidy analysis by flow cytometry on freshly isolated myocyte nuclei using an antibody against a cardiomyocyte-specific nuclear marker, pericentriolar material 1 (PCM-1), and a DNA-binding dye. As expected, approximately 30% of the total cardiac nuclei from both *Tnnc1*^WT/WT^ (Figure 5B) and *Tnnc1*^A8V/A8V^ (Figure 5E) mice were positive for the PCM-1 marker for cardiomyocyte nuclei. Cardiac nuclei were also immunolabeled with an isotype control to account for the possibility of non-specific binding of the secondary antibody (Figure 5A,D). Diploid (2n), tetraploid (4n), octoploid (8n), and hexadecaploid (16n) cardiomyocyte nuclei populations from both groups of mice were detected (Figure 5C,F). Although cardiomyocyte nuclei from *Tnnc1*^A8V/A8V^ mice tended to exhibit a slightly smaller percentage of diploid nuclei, they displayed a greater percentage of polyploid nuclei compared to *Tnnc1*^WT/WT^ mice (Figure 5G). These results suggest that differences in DNA content cannot explain the observation that *Tnnc1*^A8V/A8V^ cardiomyocyte nuclei exhibit a smaller volume (~1/2) compared to *Tnnc1*^WT/WT^.

### 4.3. Compression of Cardiomyocyte Nuclei During Contracture

The mechanical properties of cardiomyocyte nuclei are relevant to myocardial physiology and disease [15,59]. Based on our findings that *Tnnc1*^A8V/A8V^ are smaller in area and volume, and because TNNC1 could be a component of the nucleoskeleton, it is possible that *Tnnc1*^A8V/A8V^ cardiomyocyte nuclei exhibit altered deformability. We therefore measured the geometric properties (length, width, area) of cardiomyocyte nuclei during spontaneous contractures of isolated cardiomyocytes. Supplementary Material, Figure S3 shows a representative time series obtained during spontaneous contracture of one binucleated cardiomyocyte isolated from a *Tnnc1*^WT/WT^ mouse heart. Throughout the 94 s duration of recording, the cardiomyocyte shortened steadily by ~30% of its resting length (Supplementary Material, Figure S3A). As the cardiomyocyte shortened, it widened (Supplementary Material, Figure S3B), with the net result that the area measured in the optical plane was nearly constant, decreasing only slightly (Supplementary Material, Figure S3C). Both nuclei exhibited similar behavior to each other and to the cell (Supplementary Material, Figure S3D–G). The two nuclei became shorter (Supplementary Material, Figure S3D) and wider (Supplementary Material, Figure S3E) as the cardiomyocyte contracted. The area of the nuclei in the optical plane decreased during myocyte contraction relative to the resting value; the decrease was steady for one nucleus and plateaued, or slightly reversed, for the other (Supplementary Material, Figure S3F). The aspect ratios for both nuclei decreased during the contraction (Supplementary Material, Figure S3G) as expected from the changes in length and width (Supplementary Material, Figure S3D,E).

We compared compression of nuclei during spontaneous contracture of *Tnnc1*^A8V/A8V^ and *Tnnc1*^WT/WT^ cardiomyocytes by plotting nucleus length as a function of myocyte length, where all lengths were normalized to their initial values under relaxed, diastolic conditions (Supplementary Material, Figure S4). This compression ratio is proportional to the elastic moduli of the nuclei in the same cardiomyocyte, but the constant of proportionality may be different for each cardiomyocyte. *Tnnc1*^A8V/A8V^ nuclei exhibited, on average, a lower compression ratio (slope of normalized data in Supplementary Material, Figure S4C) suggesting the mutant nuclei may be stiffer, but this difference was not statistically significant, likely due to the small sample size (Supplementary Material, Figure S4D). Examination of the relationship between the mechanical compression ratio and nucleus size (Supplementary Material, Figure S4D) suggests that smaller nuclei—such as those in *Tnnc1*^A8V/A8V^ cardiomyocytes—may be more resistant to compression because the slope of the regression line in Supplementary Material Figure S4D was significantly different from 0 with multiple R^2^ = 0.953; despite that correlation, however, there are individual nuclei that diverge from that tendency. Overall, these results demonstrate that cardiomyocyte nuclei are subjected to deformation during spontaneous contractions in culture and suggest that cardiomyocyte nuclei likely undergo cyclic compression/expansion during normal cardiac function in vivo.

### 4.4. Decreased Nuclear Localization of TNNC1 in Tnnc1^A8V/A8V^ Mouse Hearts

Mounting evidence has led to the proposal that nucleocytoplasmic shuttling of molecular cargo (i.e., proteins and nucleic acids) influences nucleus size [58]. Furthermore, it has been experimentally demonstrated that nuclear import/export mechanisms are perturbed in the setting of pathological cardiomyocyte remodeling [33,60,61]. We previously showed that a fraction of TNNC1, along with other myofilament proteins, is localized within the nuclear compartment of cultured neonatal rat ventricular cardiomyocytes [62]. Therefore, we reasoned TNNC1 would also be expressed in the adult heart and that smaller *Tnnc1*^A8V/A8V^ cardiomyocyte nuclei might be associated with altered nuclear localization of mutant TNNC1.

**Figure 6:**
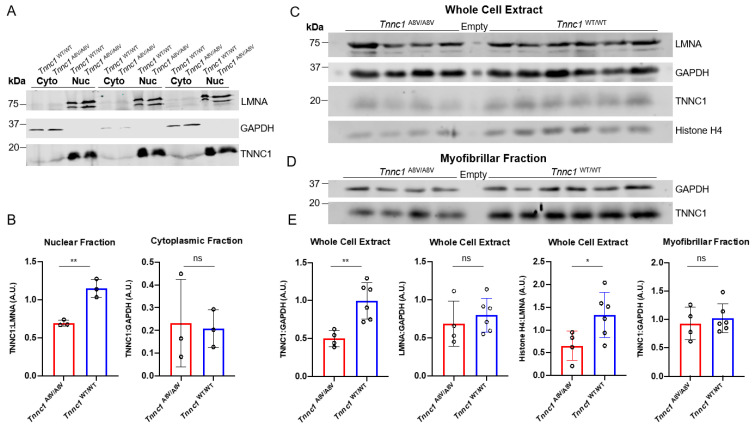
Subcellular fractionation and immunoblot analysis of mouse heart tissue. Note that cardiac nuclei were not fluorescently sorted for this experiment. (**A**) Immunoblot image of cytosolic (Cyto) and nuclear (Nuc) fractions (25 µg) from 4 to 6-month-old mice for each genotype. (**B**) Corresponding densitometric quantification of TNNC1 relative to LMNA abundance (TNNC1:LMNA) and TNNC1 relative to GAPDH abundance (TNNC1:GAPDH). *Tnnc1*^WT/WT^ (*n* = 3 hearts) and *Tnnc1*^A8V/A8V^ (*n* = 3 hearts). (**C**) Immunoblot image of whole cell extracts (40 µg each). (**D**) Immunoblot image of myofibrillar fractions (30 µg each). Empty lanes correspond to no protein loaded. *Tnnc1*^WT/WT^ (*n* = 6 hearts) and *Tnnc1*^A8V/A8V^ (*n* = 4 hearts). (**E**) Corresponding densitometric quantification of TNNC1, LMNA and Histone H4 relative to GAPDH. ** *p* < 0.005; * *p* < 0.05, unpaired Student’s *t*-test. Data are presented as mean ± SD. A.U., Arbitrary Units. ns, not significant.

**Figure 7:**
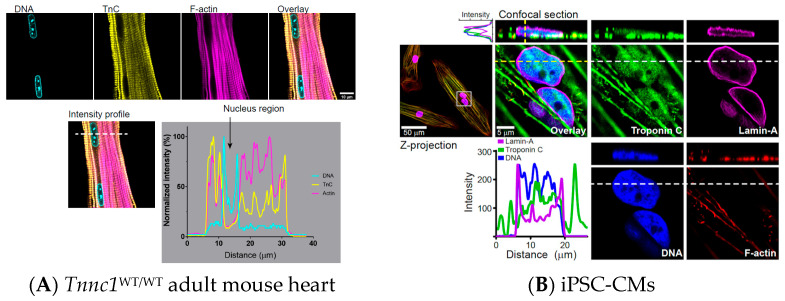
Confocal immunofluorescence microscopy imaging of (**A**) *Tnnc1*^WT/WT^ adult mouse heart tissue and (**B**) hiPSC-CMs derived from a healthy volunteer. Note lack of signal for troponin C (yellow) within adult mouse heart nuclei (DNA stain, blue) and positive signal for troponin C (green) within hiPSC-CM nuclei (blue, DNA; magenta, lamin A). Images were acquired on a Leica TCS SP8 system with 63×/1.40 NA oil-immersion objective and processed using ImageJ.

To address this possibility, we carried out crude subcellular fractionation on *Tnnc1*^WT/WT^ and *Tnnc1*^A8V/A8V^ mouse hearts followed by immunoblot analysis (Figure 6A). Purity of the fractions is indicated by enrichment of the nuclear membrane protein lamin A/C (LMNA) exclusively in nuclear fractions and glyceraldehyde 3 phosphate dehydrogenase (GAPDH) exclusively in cytosolic fractions (Figure 6A). We found TNNC1 in nuclear fractions from *Tnnc1*^WT/WT^ mouse hearts and a significantly lower relative abundance of mutant TNNC1 in *Tnnc1*^A8V/A8V^ mouse hearts (Figure 6B). There was no significant difference in the relative abundance of cytoplasmic TNNC1 between *Tnnc1*^WT/WT^ and *Tnnc1*^A8V/A8V^ mouse hearts (Figure 6B). In a separate set of experiments, we analyzed whole cell extracts and isolated myofibrils from *Tnnc1*^WT/WT^ and *Tnnc1*^A8V/A8V^ mouse hearts by immunoblotting (Figure 6C,D). We found that the relative abundance of TNNC1 in whole cell extracts of *Tnnc1*^WT/WT^ mouse hearts was significantly lower than in *Tnnc1*^A8V/A8V^, but there was no difference in myofibril preparations (Figure 6E). Because our results suggested potential differences in nucleus stiffness (Supplementary Material, Figure S4), we also quantified the expression levels of LMNA in whole cell extracts. No significant difference in the relative abundance of LMNA was observed upon comparing *Tnnc1*^WT/WT^ and *Tnnc1*^A8V/A8V^ (Figure 6E). Surprisingly, the relative abundance of histone H4 in *Tnnc1*^A8V/A8V^ mouse hearts was significantly lower compared with that in *Tnnc1*^WT/WT^ mouse hearts (Figure 6E). In contrast to our prediction, we could not detect nuclear localization of TNNC1 by immunofluorescence microscopy in adult *Tnnc1*^WT/WT^ mouse heart tissue (Figure 7A). Nuclear localization of TNNC1, however, was observed in human induced pluripotent stem cell-derived cardiomyocytes (hiPSC-CMs) derived from a healthy volunteer (Figure 7B). A secondary antibody-only control was performed to ensure that the positive staining was specific to the primary antibody against TNNC1 (Supplementary Material, Figure S5). These results suggest that a fraction of TNNC1 is localized within the nuclear compartment of mouse cardiomyocytes and that nucleocytoplasmic transport of TNNC1 may be compromised in *Tnnc1*^A8V/A8V^ mouse hearts.

## 5. Discussion

In the present study, we found that cardiomyocyte nuclei undergo marked structural remodeling in a mouse model of HCM caused by a pathogenic missense mutation in TNNC1 (Tnnc1-p.A8V). Tnnc1-p.A8V mice displayed abnormalities in cardiomyocyte nuclear morphology (i.e., smaller and rounder) without changes in ploidy and chromatin content, along with reduced nuclear localization of TNNC1 and decreased expression of histone H4. These observations raise several intriguing questions and could have important implications for myocyte biology.

Nuclear structure is controlled by a multitude of key factors, including cell size, LInker of Nucleoskeleton and Cytoskeleton (LINC) proteins, gene transcription, lipid metabolism, and nucleocytoplasmic transport [58]. One question raised in this study is precisely how a sarcomeric protein mutation (Tnnc1-p.A8V) can have such a profound impact on cardiomyocyte nuclear morphology (Figure 1, Figure 2 and Figure 3). While we did find that *Tnnc1*^A8V/A8V^ nuclei might be rounder (Figure 2D) simply because they are smaller (Figure 2 and Figure 3), it does not address why nucleus size is altered. In physiological conditions, nucleus size tends to scale with cell size in a variety of organisms and cell types, which maintains a constant N/C ratio [58]. In pathological settings, however, the N/C ratio can be disrupted—a phenomenon that is most notable in cancer cells, where nuclei often exhibit bizarre morphologies [23]. We found that the smaller area and volume of *Tnnc1*^A8V/A8V^ cardiomyocyte nuclei could not be explained by differences in cell diameter, which was used as an index for size (Figure 4). In fact, neither nucleus area, volume, nor aspect ratio was related to cardiomyocyte diameter in *Tnnc1*^WT/WT^ or *Tnnc1*^A8V/A8V^ mice (Figure 4C–E). One possible explanation for these findings is that myocyte diameter does not accurately reflect cardiomyocyte size. However, a strong correlation between myocyte length and diameter was observed (Figure 4A), and it has been previously shown that these parameters are suitable for extrapolating myocyte volume/size [63]. Most current knowledge on nucleus scaling is derived from experiments on simple model organisms (e.g., budding/fission yeast) [64,65,66], and thus another possibility is that the concept of nuclear scaling, at least in the classical sense, may not apply to cardiomyocytes. However, one group leveraged a Drosophila model system to explore the mechanisms of myocyte nuclear scaling in vivo [67]. Their results suggest that individual nuclei within a skeletal myofiber establish discrete local scaling relationships determined by global, regional, and local factors [67,68]. Furthermore, this concept of the myonuclear domain established in skeletal muscle fibers [69,70] may also apply to cardiomyocytes despite the smaller number of nuclei [56,71].

If individual nuclei within multinucleated skeletal muscle fibers scale with nuclear activity rather than cell size per se, it stands to reason that a similar mechanism probably operates in multinucleated cardiomyocytes, and thus cardiomyonuclear domain size may be physiologically relevant [71]. The predominance of binucleated cardiomyocytes in small rodents (mouse, rat, rabbit) [56], as observed in the present study (Supplementary Material, Figure S1), allowed evaluation of the relationship between paired nuclei within individual cells. In the binucleated cardiomyocytes examined, we found that the sizes of paired nuclei were correlated, but that one nucleus was larger than the other in both *Tnnc1*^WT/WT^ and *Tnnc1*^A8V/A8V^ mice (Supplementary Material, Figure S2). These results are consistent with the suggested coordination between scaling of individual nuclei within a skeletal myofiber based on local factors (e.g., myoplasmic domain size) [67]. In further support of this concept, it was previously shown that nuclear volume correlated with local cytoplasmic volume in multinucleated yeast cells [64]. Despite the tendency for distinct nuclei in multinucleated cells to exhibit different sizes based on various factors, the biological significance of this emerging trend is unclear. It seems plausible to speculate that the larger nucleus in a binucleated cardiomyocyte indicates greater nuclear activity relative to the smaller nucleus. A previous study found that only one of the two nuclei in binucleated cardiomyocytes was typically associated with nuclear Ca^2+^ signaling and expression of certain regulatory proteins [72]. Based on these observations, the authors proposed that one nucleus might be dominant in activity while the other is ‘dormant’ [72]. Interestingly, in contrast to rodents, human ventricular myocardium with its lower metabolic load is predominantly composed of mononucleated cardiomyocytes, with approximately only 25% binucleated cardiomyocytes [73]. The potential physiological relevance of species-specific differences in the proportion of mono- and multi-nucleated cardiomyocytes remains uncertain at this time, but one could speculate that it might reflect an evolutionary mechanism for adaptation to cellular metabolic load and stress.

Since there was no significant relationship between cell size and nucleus size, we reasoned that lower DNA content (e.g., 2-fold lower ploidy on average) might explain why *Tnnc1*^A8V/A8V^ cardiomyocyte nuclei are smaller. In contrast to this expectation, cardiomyocyte nuclei isolated from *Tnnc1*^A8V/A8V^ mice did not display lower ploidy (Figure 5); thus, we do not believe this possibility can explain the smaller (52%) nucleus volumes (Figure 3). Furthermore, accumulating evidence indicates that DNA content is not a significant determinant of nucleus size [58]. Experiments in fission yeast demonstrated that a 16-fold increase in DNA content via genetic manipulation had no apparent effect on nucleus size [64]. In response to stress or injury, mammalian cardiomyocytes can increase their DNA content without undergoing mitosis, leading to polyploidy [74,75]. This process is commonly referred to as ‘endoreduplication’ and serves as a striated muscle growth mechanism. It is therefore not surprising that we observed a higher percentage of polyploid cardiomyocyte nuclei in *Tnnc1*^A8V/A8V^ mice exhibiting HCM (Figure 5). One likely outcome of *Tnnc1*^A8V/A8V^ cardiomyocyte nuclei having significantly smaller volumes without a commensurate reduction in ploidy is that chromatin is condensed to a greater degree (e.g., increased heterochromatin). Our findings also have notable parallels to a study on mechanotransduction in Drosophila skeletal muscle fibers [76]. The authors reported that deletion of specific proteins in the LINC complex resulted in significantly smaller myonuclei as well as increased (and variable) DNA content in single myofibers. Furthermore, the Drosophila LINC mutants exhibited significantly reduced expression of barrier-to-autointegration factor (BAF) and, remarkably, troponin C. The authors subsequently proposed that mechanotransduction through the LINC complex, mediated by BAF, controls synchronization of cell cycle progression [76]. In light of these results along with our present findings, it would appear that dysregulation of myofilament proteins may play a role in nuclear mechanotransduction [77] and DNA endoreplication in striated muscle cells.

External forces emanating from the extracellular space are ultimately propagated to the nuclear membrane through elaborate cytoskeletal networks, which form the basis of nuclear mechanotransduction [17]. In contrast to skeletal myocyte nuclei, which are positioned at the periphery of the myofiber and can be affected by contractile activity [78,79,80], cardiomyocyte nuclei are embedded between contractile myofibrils arranged in a 3-D myofilament lattice (Figure 1, Figure 2 and Figure 3). With this structural relationship in mind, it seems reasonable to expect that cardiomyocyte nuclei are mechanically compressed during the systolic (contractile) phase of each cardiac cycle. Indeed, this concept was initially proposed in the 1970s based on experimental data that revealed a relationship between sarcomere length and myocardial nuclear membrane changes [26,81]. Although we showed *Tnnc1*^WT/WT^ and *Tnnc1*^A8V/A8V^ cardiomyocyte nuclei are longitudinally compressed in a non-physiological condition (i.e., spontaneous contracture rather than the systolic-diastolic, cardiac mechanical cycle) (Supplementary Material, Figures S3 and S4), our results are nevertheless in excellent agreement with literature reporting on dynamic deformation of myocyte nuclei during contraction [18,27,28]. In the present study, we did not find a significant difference in the mechanical properties of cardiomyocyte nuclei between *Tnnc1*^WT/WT^ and *Tnnc1*^A8V/A8V^ (Supplementary Material, Figure S4C), but there was a statistically significant relationship with nucleus size when the two samples were combined (Supplementary Material, Figure S4D). If the forces generated by *Tnnc1*^WT/WT^ and *Tnnc1*^A8V/A8V^ cardiomyocytes were equal during the measurements—or if the force of *Tnnc1*^A8V/A8V^ cardiomyocytes was larger, which is possible given the increased Ca^2+^-sensitivity associated with TNNC1 [42,43,44]—then a smaller compression ratio would indicate increased nuclear stiffness, i.e., a larger elastic modulus (Supplementary Material, Figure S4). If we consider that cardiomyocyte nuclear stiffness has been shown to positively correlate with chromatin condensation [27], our experimental results in Figure 5 (ploidy analysis) are congruent with the trend of higher nuclear stiffness of the smaller nuclei in *Tnnc1*^A8V/A8V^ cardiomyocytes (Supplementary Material, Figure S4). Furthermore, a previously reported study using elongated endothelial cells supports this possibility, whereby a reduction in nuclear volume was associated with increased nuclear stiffness and chromatin condensation [82]. The extracellular matrix and cytoskeleton may also influence mechanical properties of the nucleus [15,77]. Interstitial fibrosis (extracellular matrix factor) and myofibrillar disarray (cytoskeleton factor), which are characteristic of HCM, were previously identified in *Tnnc1*^A8V/A8V^ mice [42]. Such factors could have partially contributed to the structural abnormalities in *Tnnc1*^A8V/A8V^ cardiomyocyte nuclei. Notwithstanding, there is strong evidence that cardiomyocyte nuclei are dynamically deformed during contraction. Alterations in contractility associated with HCM may therefore have a direct impact on nuclear deformation and genome regulation.

### 5.1. Implications for Sarcomeric Cardiomyopathies

From a classical perspective on HCM pathophysiology, it is proposed that sarcomere dysfunction triggers mechanical and Ca^2+^-induced signaling pathways that ultimately culminate in altered gene expression and hypertrophic remodeling of the myocardium [83]. However, the underlying mechanisms linking contractile dysfunction with activation or repression of gene transcription remain largely enigmatic. We have identified extensive transcriptome differences in *Tnnc1*^A8V/A8V^ mouse hearts compared to *Tnnc1*^WT/WT^ by bulk RNA sequencing [45]. Though purely speculative, it would not be entirely unfounded to suggest that the abnormal nuclear morphology observed in *Tnnc1*^A8V/A8V^ mice could partially contribute to the global changes in steady-state mRNA expression in *Tnnc1*^A8V/A8V^ mice. The concept that myocardial stretch could be directly coupled to chromatin reorganization and hypertrophic growth initiation was initially proposed by Bloom et al. [84]. A seminal study showed that application of external mechanical load induced chromatin stretching and upregulation of a reporter transgene in cultured cells [19]. The authors provided further insight into the structural basis by proposing a model in which externally applied force is sensed by integrins and propagated through the cytoskeleton and LINC complex to the nuclear membrane interior, leading to BAF- and heterochromatin protein 1-mediated stretching of flanking chromatin [19]. Disruption of LINC complex proteins or plating cardiomyocytes on a stiff substrate impacts intranuclear strain [18]. Furthermore, another study found that modulation of cardiac myosin-II contractility with omecamtiv mecarbil or a mavacamten analog in beating embryonic chick hearts rapidly impacted nuclear structure as well as DNA damage [21].

We previously reported that desmin protein expression is upregulated in *Tnnc1*^A8V/A8V^ myofibrils [85], and a balance between desmin and microtubules has been shown to be essential for cardiomyocyte nuclear homeostasis and sarcomere contractility [25]. In addition, one of the most significant changes in transcription in *Tnnc1*^A8V/A8V^ hearts is for integrin-linked kinase (ILK) pathway signaling molecules, a pathway known to mediate hypertrophic responses to mechanical stress [45]. Interestingly, abnormal nuclear morphology was observed previously in cardiac histological sections from human subjects with DCM caused by TTN truncating mutations, although it is difficult to draw conclusions from this finding without proper control tissue sections [86]. Furthermore, it has been reported that loss of GSK 3 in a mouse model leads to DCM and an increase in cardiomyocyte nuclear enlargement [87]. In contrast to our findings, a previous study reported increased cardiomyocyte nucleus area in HCM caused by an MYBPC3 mutation [88]. The reason for this difference is unclear, but there are a number of potential explanations. First, due to the progressive nature of myocardial remodeling and the extent of myocyte disarray, hypertrophy, and fibrosis in cardiomyopathy in each experimental model, the specific timepoint used for analysis of nuclear dimensions could impact the final results. Second, there could be mutation or gene-specific effects, especially in light of the observation of nuclear-localized TNNC1. A final possible explanation could be due to technical differences in identifying cardiomyocyte nuclei versus non-myocyte nuclei. In contrast to the aforementioned study, which only examined tissue sections, most of our image analyses were conducted on isolated cardiomyocytes to eliminate the possibility of quantifying nuclear dimensions of non-myocytes, in addition to the initial analyses of myocardial tissue sections.

Taken together, these results suggest a link between altered cardiomyocyte contractility and nuclear architecture via mechanotransduction. Importantly, abnormalities in cardiomyocyte nuclear morphology and mechanics reported herein could be due to a combination of decreased nuclear expression of TNNC1/histone H4, desmin upregulation, interstitial fibrosis, and sarcomere hypercontractility in this *Tnnc1*^A8V/A8V^ mouse model of HCM.

### 5.2. Implications of Sarcomeric and Cytoskeletal Proteins in the Cardiomyocyte Nucleus

It is now well documented that several proteins classically associated with functions in the cell cytoplasm (e.g., actin and myosins) may also serve crucial roles in the nucleus [89,90,91]. We previously reported that a fraction of TNNC1 was present within nuclei of neonatal rodent ventricular cardiomyocytes [62], but it was unclear whether this phenomenon occurs in adult cardiomyocyte nuclei. Furthermore, the biological significance of cardiomyocyte nuclear troponins remains largely unknown. In the present study, we identified TNNC1 in adult mouse cardiac nuclear fractions and found that *Tnnc1*^A8V/A8V^ mouse hearts exhibited a significant reduction in the relative abundance of nuclear-localized TNNC1 by immunoblotting (Figure 6A,B). However, we were unable to detect nuclear localized TNNC1 in fixed myocardial tissue by immunofluorescence staining (Figure 7A). On the other hand, examination of iPSC-CMs revealed nuclear localization of TNNC1 (Figure 7B). Technical limitations may explain this discrepancy because nuclear antigens can be difficult to detect in fixed tissues due to masking of the antigen by DNA/chromatin-associated proteins, thereby compromising immunoreactivity, or antibody penetration issues. Alternatively, it is possible that the presence of TNNC1 in the nuclear fractions is merely due to myofilament contamination during subcellular fractionation. If it turns out that TNNC1 is only expressed in immature or fetal cardiomyocyte nuclei and not in the adult heart, pathogenic mutations, including A8V, could still disrupt nuclear structure and function early in development and underlie the pathophysiology of cardiomyopathy. Nevertheless, our results raise several important questions and may have implications for the molecular pathogenesis of HCM caused by pathogenic variants in TNNC1.

Intracellular Ca^2+^ not only plays a key role in regulating cardiac contractility, but it is also thought to be involved in signaling pathways controlling gene expression, commonly referred to as excitation-transcription coupling [92]. Importantly, defects in nucleus structure and nuclear Ca^2+^ signaling have been proposed to be early pathological events in the setting of pressure overload-induced cardiac hypertrophy and heart failure [93]. Considering TNNC1 is a member of the EF hand family of Ca^2+^-binding proteins, it is possible that TNNC1 may have a role in Ca^2+^-dependent aspects of excitation-transcription coupling in the cardiomyocyte nucleus, perhaps through buffering intra/perinuclear Ca^2+^ or regulating chromatin organization. Of potential relevance, it has been suggested that cardiac troponin T (TNNT2) might function as an epigenetic factor in hiPSC-CMs [94]. Similarly to our findings, the authors of this study found altered nuclear localization of mutant TNNT2 in a hiPSC-CM model of DCM [94]. Since TNNT2 and TNNC1 are two of the three subunits that form the troponin complex in the cardiac thin filament [95,96,97,98,99], and we identified nuclear localization of TNNC1 in hiPSC-CMs (Figure 7), these troponin subunits might cooperate in epigenetic regulation of gene transcription in cardiomyocytes. This theory would be consistent with the evidence regarding the nuclear functions of other classical cytoskeletal proteins (nuclear actin and myosin), which have been implicated in a force-generating mechanism responsible for regulating chromatin organization [100]. The troponin complex has also been demonstrated to enhance contractility through a direct interaction with myosin [101], a property that could further modulate any potential role in regulation of chromatin organization. Further investigations are certainly warranted to elucidate the precise function(s) of nuclear-localized TNNC1. If it turns out that TNNC1 has demonstrable roles in cardiomyocyte nuclei, it might help to explain why human TNNC1-linked cardiomyopathies are typically associated with a relatively poor prognosis [102,103] and why some pathogenic variants in TNNC1 cannot be explained by perturbed myofilament mechanical properties [104].

Another question that we sought to address is why nuclear localization of TNNC1 in *Tnnc1*^A8V/A8V^ mice is decreased (Figure 6). TNNC1 does not contain a canonical nuclear localization signal [105], so it is predicted that TNNC1 is not likely to localize to the nucleus on its own [106]; thus, we do not suspect the A8V mutation alters signal-dependent nuclear import. However, because cardiac troponin I (TNNI3) contains a nuclear localization signal [73,107] and has been detected in adult cardiomyocyte nuclei [108], it is possible that disruption of this well-established binary interaction reduces nuclear import of TNNC1. Additionally, structural remodeling of the nucleus, as observed in the present study, might underlie reduced nuclear localization of TNNC1 *Tnnc1*^A8V/A8V^ mouse hearts. Cardiomyocyte nuclear pore complexes display remarkable structural plasticity to dynamically regulate import and export in response to altered stress [109]. Hypertrophied myocytes (e.g., HCM) demand increased export of genetic material from the nucleus to support protein synthesis in the sarcoplasm and fuel myocardial growth [110]. It was previously demonstrated that the nuclear pore complexes of hypertrophied and failing cardiomyocytes undergo remodeling to favor nuclear export and suppress nuclear import activity [60,61]. It was shown that remodeling of the nuclear architecture and alterations in nucleocytoplasmic shuttling were early events in hypertrophic growth, preceding the development of heart failure [61]. Thus, decreased nuclear localization of TNNC1 in *Tnnc1*^A8V/A8V^ mouse hearts may be due to smaller nuclear dimensions. Another striking yet puzzling observation in the present study is the decreased expression of histone H4 in *Tnnc1*^A8V/A8V^ mouse hearts revealed by immunoblotting (Figure 6C). The potential functional significance of this finding is unclear, but histone H4 is a highly conserved protein classically responsible for regulating chromatin compaction. Furthermore, a study on *C. elegans* suggests that histone H4 may also serve a role in regulating mitochondrial activity and organismal longevity [111]. Determining the potential impact of altered histone H4 levels in the heart is certainly worth future investigation.

## 6. Conclusions

In summary, our data reveal that a mouse model of HCM caused by a sarcomeric protein mutation is linked to abnormalities in cardiomyocyte nuclear morphology. One limitation in our study is that the experimental design did not permit assessment of whether morphological remodeling of the nuclei precedes the development of HCM; hence, it is unclear whether this observation represents a primary driver or secondary consequence of the Tnnc1-p.A8V mutation. Nevertheless, we propose that sustained myocardial hypercontractility in *Tnnc1*^A8V/A8V^ mice, caused by an increased Ca^2+^ binding affinity to the N-lobe of TNNC1, is coupled to structural remodeling of cardiomyocyte nuclei, thereby driving the progression of HCM. It is therefore no mystery why pathogenic mutations in TNNC1 can cause human cardiomyopathy [43,102,103,112].

## Data Availability

The original contributions presented in this study are included in the article/Supplementary Materials. Further inquiries can be directed to the corresponding author.

## Supplementary Materials

The following supporting information can be downloaded at: https://www.mdpi.com/article/10.3390/jcdd12110449/s1, Figure S1: The number of nuclei per cardiomyocyte (nuclearity) was counted in isolated cardiomyocytes. Tnnc1WT/WT (*n* = 67), Tnnc1A8V/A8V (*n* = 29), and C57BL/6J (*n* = 19). There was no significant difference in nuclearity between the three groups; *p* > 0.05, χ2 test; Figure S2: In relaxed, binucleated cardiomyocytes, the sizes of paired nuclei are correlated, but one nucleus is significantly larger than the other. Nucleus #1 (abscissa in all panels) was defined as the nucleus with the larger cross-sectional area, and thus nucleus #2 (ordinate in all panels) was the smaller of the two. Correlation (multiple R^2^ > 0.934) between the areas (A) and volumes (B) of paired nuclei in single, isolated Tnnc1A8V/A8V (red squares) and Tnnc1WT/WT (blue circles) cardiomyocytes. Lines in both (A) and (B) are linear least squares regressions constrained to pass through the origin; note that the volume data for Tnnc1A8V/A8V and Tnnc1WT/WT were combined for regression analysis (black line in B) because of the small sample size. (C) Relationship between nucleus shapes (aspect ratio; length:width ratios) for paired nuclei in single, isolated Tnnc1A8V/A8V (red squares and solid line) or Tnnc1WT/WT (blue circles and solid line) cardiomyocytes. Lines are from nonlinear regression analyses where the regression was a straight line constrained to pass through (1, 1). Slopes were <1.0 for all regressions shown in panels A–C and are provided in Results; Figure S3: Time course of spontaneous contracture for a single, binucleated Tnnc1WT/WT cardiomyocyte. Cell dimensions: length (A); diameter (B); and area (C). Nucleus: length (D); width (E); area (F); and shape (aspect ratio; length:width ratio) (G). In panels D–F, nucleus #1 (larger area at time = 0) data points are garnet and nucleus #2 (smaller area at time = 0) data points are gold; Figure S4: Cardiomyocyte nuclei are compressed during spontaneous contraction. Nucleus length (the dimension along the contractile axis of the cardiomyocyte) was plotted as a function of cell length (both lengths normalized to the initial, relaxed values) for *n* = 3 nuclei from two Tnnc1A8V/A8V cardiomyocytes (A) and *n* = 7 nuclei from 4 Tnnc1WT/WT cardiomyocytes (B) during spontaneous contractions (as in Fig. 6). Each nucleus is plotted in a different color. Lines (same color as points) are from nonlinear regression analysis on data where the cell had shortened <20%; the regression was a straight line constrained to pass through (1, 1). The unity line (black dotted line) is also included for reference in panels A and B. (C) Boxplot summary of regression slopes from panels A (red) and B (blue). Data are presented as the median with 1st and 3rd quartile ranges. The slopes of Tnnc1A8V/A8V and Tnnc1WT/WT nuclei in this small sample were not statistically different (Welch Two Sample *t*-test, *p* > 0.05). The slopes were ~1 for Tnnc1WT/WT nuclei, while slopes were <1 for most Tnnc1A8V/A8V nuclei, indicating that the nuclei within HCM cardiomyocytes are relatively resistant to compression; Figure S5: Secondary antibody only (no primary antibody) control for confocal imaging of TNNC1 in hiPSC-CMs. Note lack of signal in the secondary antibody only condition for TNNC1. Images were acquired on a Leica TCS SP8 system with a 40×/0.7 dry objective and processed using ImageJ. Green, TNNC1; Red, F-actin; Blue, DNA.
