# Supplementary material for: Cardiomyocyte Nuclear Pleomorphism in a Mouse Model of Inherited Hypertrophic Cardiomyopathy
Source: J Cardiovasc Dev Dis. 2025 Nov 19;12(11):449. doi: 10.3390/jcdd12110449 (PMC12653334; doi:10.3390/jcdd12110449)
Supplement: Supplementary file 1 [file jcdd-12-00449-s001.zip › jcdd-3906772-supplementary.pdf]

# SUPPLEMENTARY MAATERIAL

*Article*

# Cardiomyocyte Nuclear Pleomorphism in a Mouse Model of Inherited Hypertrophic Cardiomyopathy

**Jamie R. Johnston [1,*,†], Isabella Leite Coscarella [1,‡], Carson L. Rose [2,§], Yun Shi [2,‖], Hosna Rastegarpouyani [3,¶], Karissa M. Dieseldorff Jones [1,**], Jennifer M. Le Patourel [2], Feyikemi Ogunfuwa [2,††], Adriano S. Martins [1], Kathryn M. Crotty [2,‡‡], Katherine M. Ward Molla [2], Tyler R. Reinoso [2,§§], Taylor L. Waldmann [2,‖‖], Jerome Irianto [1], Yue Julia Wang [1], Lili Wang [4], Björn C. Knollmann [4], Jose R. Pinto [1] and Prescott Bryant Chase [2]**

1 Department of Biomedical Sciences, College of Medicine, Florida State University, Tallahassee, FL 32306, USA
2 Department of Biological Science, Florida State University, Tallahassee, FL 32306, USA; yun.shi@duke.edu (Y.S.); tylerreinoso@gmail.com (T.R.R.); taylorwaldmann@ufl.edu (T.L.W.); chase@bio.fsu.edu (P.B.C.)
3 Institute for Molecular Biophysics, Florida State University, Tallahassee, FL 32306, USA
4 Vanderbilt Center for Arrhythmia Research and Therapeutics, Vanderbilt University School of Medicine, Nashville, TN 37232, USA
* Correspondence: jrj15c@med.fsu.edu
† Current address: College of Medicine, Florida State University, Tallahassee, FL 32306, USA.
‡ Current address: School of Medicine, Johns Hopkins University, Baltimore, MD 21287, USA.
§ Current address: Pediatrics, The Children's Hospital at Montefiore, Bronx, NY 10467, USA.
‖ Current address: Pediatrics, Duke University Medical Center, Durham, NC 27710, USA.
¶ Current address: David Geffen School of Medicine, University of California, Los Angeles, CA 90095, USA.
** Current address: St. Jude Children's Research Hospital, Memphis, TN 38105, USA.
†† Current address: HCA Florida Orange Park Hospital, Orange Park, FL 32073, USA.
‡‡ Current address: Molecular and Systems Pharmacology, Emory University, Atlanta, GA 30322, USA.
§§ Current address: College of Medicine, Drexel University, Philadelphia, PA 19129, USA.
‖‖ Current address: College of Medicine, University of Florida, Gainesville, FL 32610, USA.

**Figure S1**

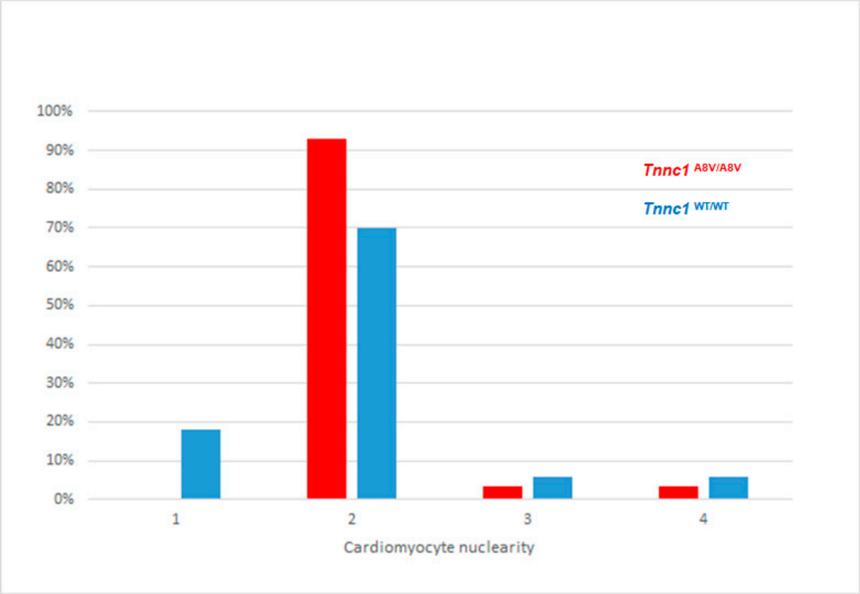


**Figure S1.** The number of nuclei per cardiomyocyte (nuclearity) was counted in isolated cardiomyocytes. *Tnnc1*$^{\mathrm{WT/WT}}$ ($n$ = 67), *Tnnc1*$^{\mathrm{A8V/A8V}}$ ($n$ = 29), and C57BL/6J ($n$ = 19). There was no significant difference in nuclearity between the three groups; $P > 0.05$, $\chi^2$ test.

**Figure S2**

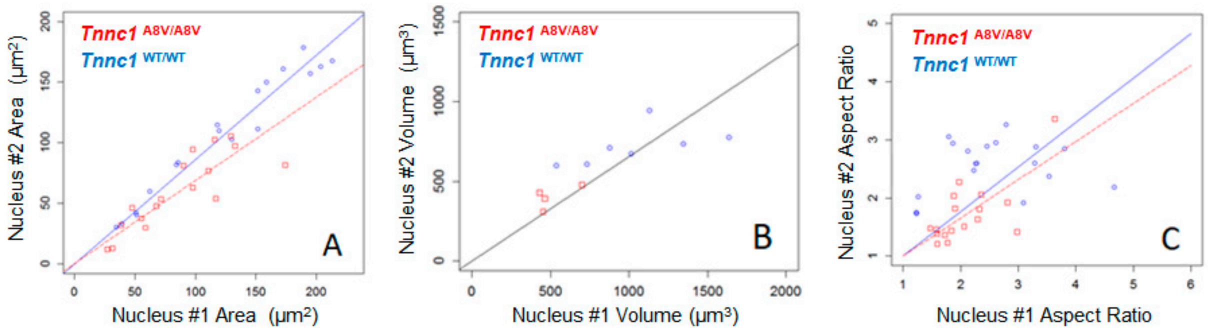


**Figure S2.** In relaxed, binucleated cardiomyocytes, the sizes of paired nuclei are correlated, but one nucleus is significantly larger than the other. Nucleus #1 (abscissa in all panels) was defined as the nucleus with the larger cross-sectional area, and thus nucleus #2 (ordinate in all panels) was the smaller of the two. Correlation (multiple $R^2 > 0.934$) between the areas (A) and volumes (B) of paired nuclei in single, isolated *Tnnc1*$^{A8V/A8V}$ (red squares) and *Tnnc1*$^{WT/WT}$ (blue circles) cardiomyocytes. Lines in both (A) and (B) are linear least squares regressions constrained to pass through the origin; note that the volume data for *Tnnc1*$^{A8V/A8V}$ and *Tnnc1*$^{WT/WT}$ were combined for regression analysis (black line in B) because of the small sample size. (C) Relationship between nucleus shapes (aspect ratio; length:width ratios) for paired nuclei in single, isolated *Tnnc1*$^{A8V/A8V}$ (red squares and solid line) or *Tnnc1*$^{WT/WT}$ (blue circles and solid line) cardiomyocytes. Lines are from nonlinear regression analyses where the regression was a straight line constrained to pass through (1, 1). Slopes were < 1.0 for all regressions shown in panels A – C and are provided in Results.

**Figure S3**

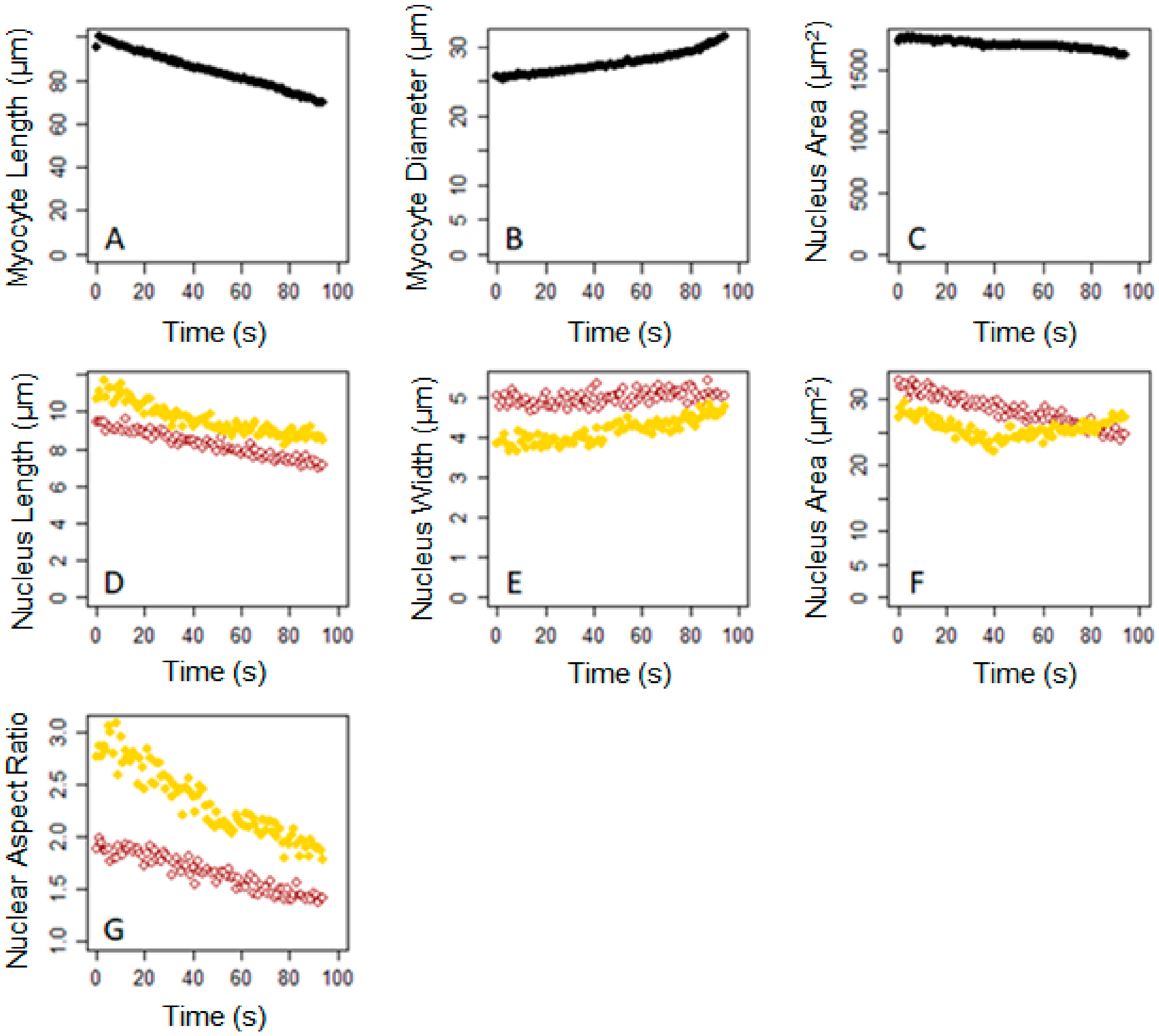


**Figure S3.** Time course of spontaneous contracture for a single, binucleated *Tnnc1*$^{WT/WT}$ cardiomyocyte. Cell dimensions: length (A); diameter (B); and area (C). Nucleus: length (D); width (E); area (F); and shape (aspect ratio; length:width ratio) (G). In panels D – F, nucleus #1 (larger area at time = 0) data points are garnet and nucleus #2 (smaller area at time = 0) data points are gold.

**Figure S4**

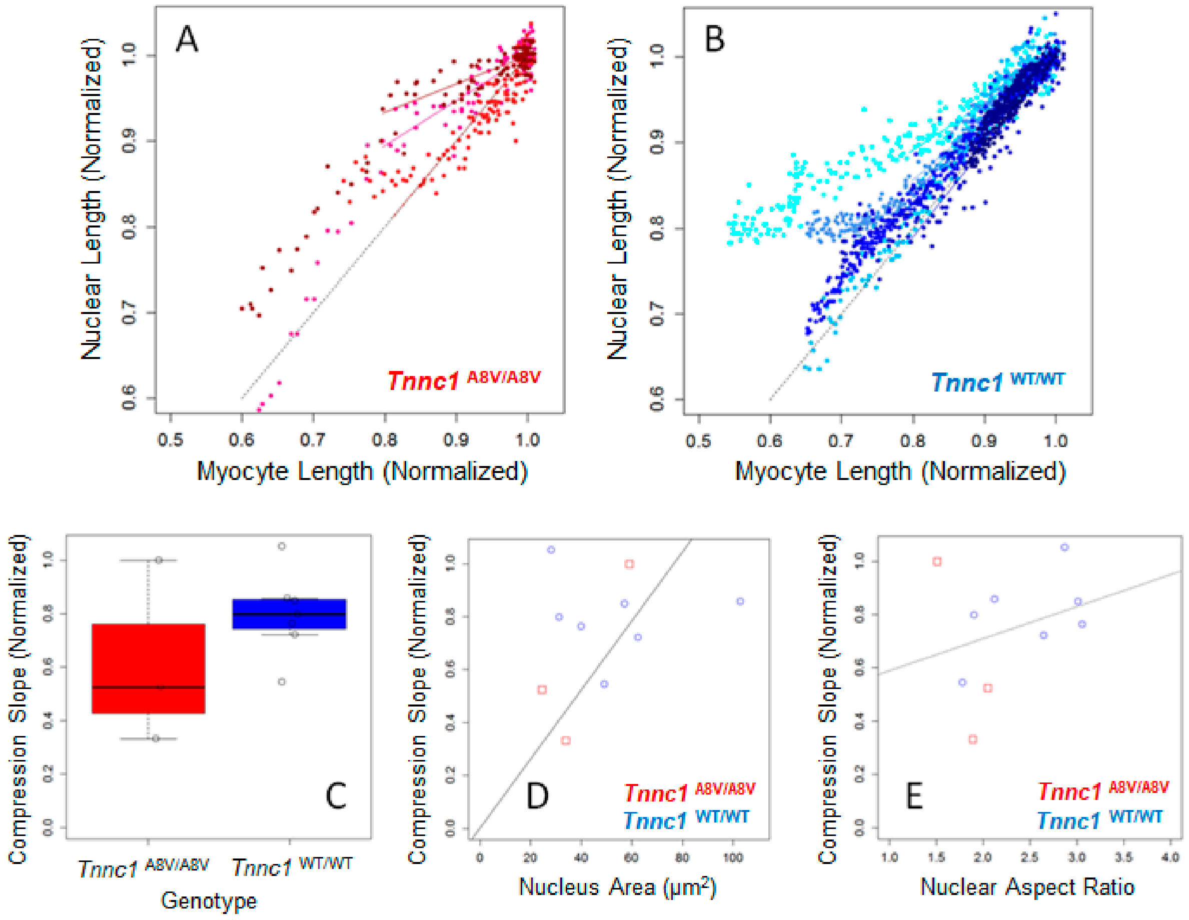


**Figure S4.** Cardiomyocyte nuclei are compressed during spontaneous contraction. Nucleus length (the dimension along the contractile axis of the cardiomyocyte) was plotted as a function of cell length (both lengths normalized to the initial, relaxed values) for *n* = 3 nuclei from two *Tnnc1*$^{A8V/A8V}$ cardiomyocytes (A) and *n* = 7 nuclei from 4 *Tnnc1*$^{WT/WT}$ cardiomyocytes (B) during spontaneous contractions (as in Figure 6). Each nucleus is plotted in a different color. Lines (same color as points) are from nonlinear regression analysis on data where the cell had shortened < 20%; the regression was a straight line constrained to pass through (1, 1). The unity line (black dotted line) is also included for reference in panels A and B. (C) Boxplot summary of regression slopes from panels A (red) and B (blue). Data are presented as the median with 1$^{st}$ and 3$^{rd}$ quartile ranges. The slopes of *Tnnc1*$^{A8V/A8V}$ and *Tnnc1*$^{WT/WT}$ nuclei in this small sample were not statistically different (Welch Two Sample t-test, $P > 0.05$). The slopes were ~ 1 for *Tnnc1*$^{WT/WT}$ nuclei, while slopes were < 1 for most *Tnnc1*$^{A8V/A8V}$ nuclei, indicating that the nuclei within HCM cardiomyocytes are relatively resistant to compression.

**Figure S5**

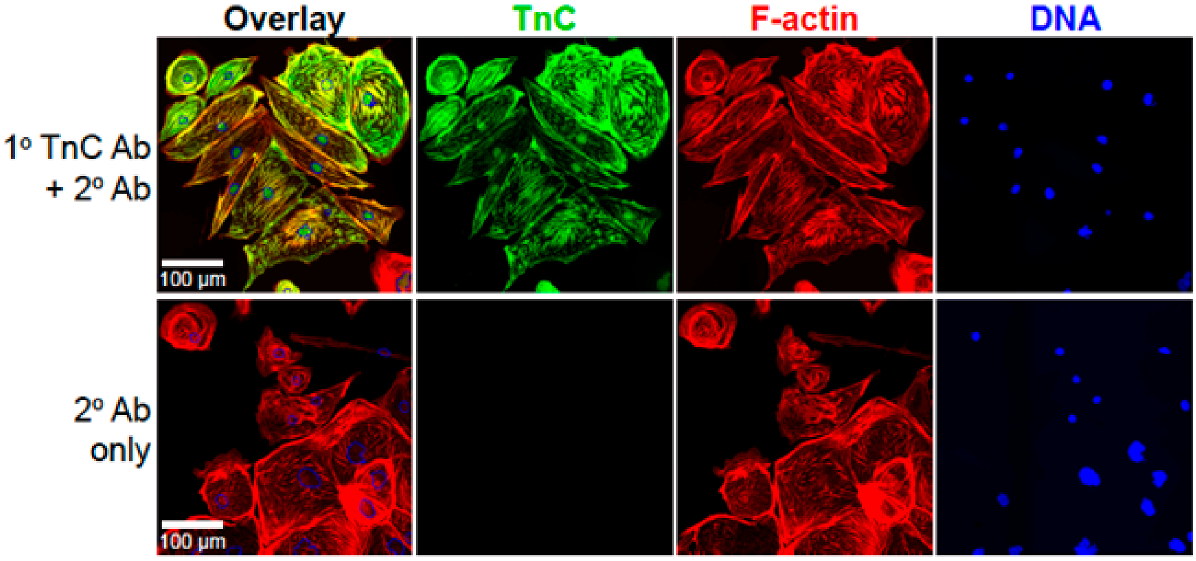


**Figure S5.** Secondary antibody only (no primary antibody) control for confocal imaging of TNNC1 in hiPSC-CMs. Note lack of signal in the secondary antibody only condition for TNNC1. Images were acquired on a Leica TCS SP8 system with a 40x/0.7 dry objective and processed using ImageJ. Green, TNNC1; Red, F-actin; Blue, DNA.
